# Commentary: From ‘sense of number’ to ‘sense of magnitude’ – The role of continuous magnitudes in numerical cognition

**DOI:** 10.3389/fpsyg.2017.00652

**Published:** 2017-05-03

**Authors:** Luca Rinaldi, Luisa Girelli

**Affiliations:** ^1^Department of Psychology, University of Milano-BicoccaMilan, Italy; ^2^NeuroMI, Milan Center for NeuroscienceMilan, Italy

**Keywords:** sense of number, sense of magnitude, statistical learning, motor system, sensorimotor experience

Insofar, the idea that the human brain is hardwired with the ability to quickly understand, approximate, and manipulate discrete numerical quantities (i.e., the so-called “number sense”; Dehaene, 1997) has received strong support from empirical research and has helped lay the foundations for mainstream theoretical frameworks of numerical cognition (e.g., Feigenson et al., 2004). It is only recently, however, that some studies have started to challenge this prevailing view, by suggesting that processing continuous magnitudes may not only be more automatic, but may also have earlier ontogenetic roots than processing discrete numerosities (e.g., Gebuis and Reynvoet, 2012; Leibovich and Ansari, 2016). Along these lines, a considerable effort to support the existence of such a “sense of magnitude” and to gather together this scattered empirical evidence into a unified theory was done by Leibovich et al. (Leibovich et al., 2016; see also Henik et al., 2017). In their theoretical model, in fact, Leibovich et al. argued that humans are born with the innate ability to recognize, process and distinguish between continuous magnitudes, and not discrete numerosities. The ability to process numerosities would thus not be innate, but rather acquired via experience. In particular, because discrete and continuous magnitudes usually correlate in the surrounding environment (e.g., the more the candies, the more the space occupied on the table), the “number sense” would develop only once this association has been assimilated and understood.

Leibovich et al. suggest that infants can learn the natural correlation between number and continuous magnitudes through “statistical learning” (e.g., Frost et al., 2015). But does the learning of this correlation simply rely on a “mere” exposure to natural scene statistics? Statistical learning implies the extraction of distributional properties from sensory input across time and space to generate and update internal representations (Frost et al., 2015). Yet, it is worth specifying that theories of statistical learning have emerged primarily in the language domain and, as such, they do not emphasize the contribution of sensorimotor transformations to internal representations. From a motor cognition standpoint, indeed, perception, and action processes are functionally intertwined. Hence, not only *perceiving* but also *acting* may help us to understand the surrounding environment and, in particular, to extract from it information about magnitude. In fact, there is plenty of evidence suggesting a primary role of sensorimotor experience in numerical and magnitude processing (e.g., Andres et al., 2008). Accordingly, we believe that the “sense of magnitude” theory should acknowledge the unique contribution of the sensorimotor system in picking up and implicitly assimilating the statistical properties related to mapping size to numerosities in our environment.

The view that time, space, number, size, speed, and other magnitudes are coupled metrics for action is certainly not new (e.g., Walsh, 2003). Ocular scanning, motor reaching, grasping, and object manipulation are indeed basic foundational bricks for the development of magnitude processing (Bueti and Walsh, 2009). Accordingly, information about magnitude would be processed by a generalized system located in the parietal cortex because of the need to encode quantities for action (Walsh, 2003). Critically, in phylogenetic terms, the capacity to manipulate discrete quantities may have evolved from the abilities in processing continuous quantities for action (Bueti and Walsh, 2009). Similarly, it has been very recently suggested that over development not only language but also object exploration may facilitate the differentiation of a generalized magnitude system into distinct quantitative dimensions (Newcombe et al., 2015). Support for this position may come from two independent lines of evidence.

First, we pinpoint that, in many ecological situations, exploring visually more items or greater surfaces may require more fixations and saccades than exploring less items or smaller surfaces (e.g., Watson et al., 2007; Gandini et al., 2008; see Graphical Abstract 1a). As a consequence, a direct correlation exists between the “size” of the visual scene and the oculomotor involvement required to explore it, with the brain that may learn to solve “more than–less than” comparisons by computing the amount of sensorimotor resources involved in the task at hand. Interestingly, prohibiting eye movements has a very negative impact on enumeration of large numerical sets, indicating that oculomotor resources are functional to numerical processing (Watson et al., 2007).

**Graphical Abstract 1:**
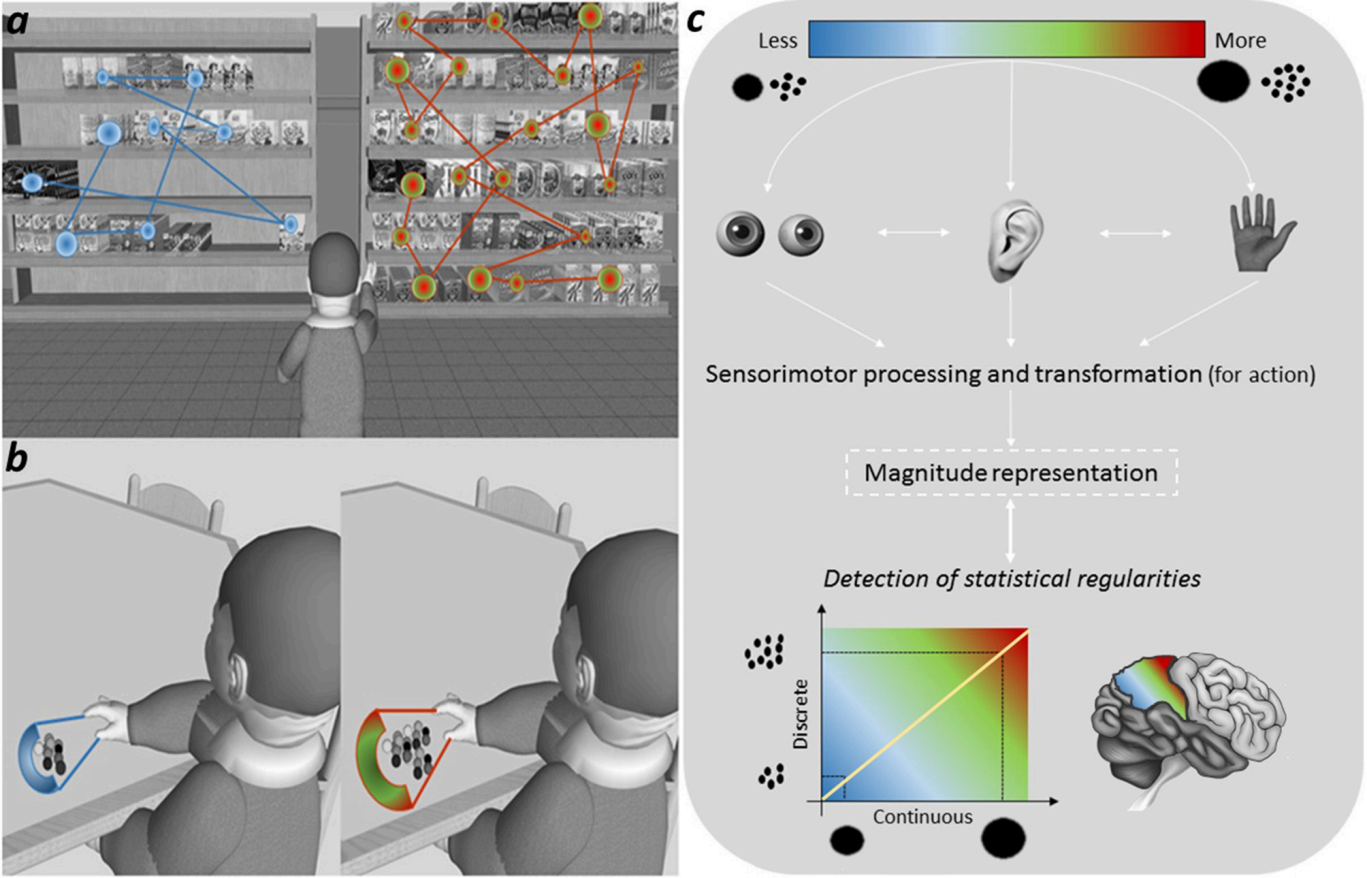
**The active involvement of the sensorimotor system in learning magnitude-related statistics of natural scenes**. The natural correlation between numerosities and continuous magnitudes may be learned not only through passive viewing, but also from active exploration. For instance, exploring visually more items or greater surfaces typically require more oculomotor resources (e.g., fixations and saccades) than exploring less items or smaller surfaces **(a)**. Similarly, action programming and online control of manual grasping significantly vary as a function of the object size **(b)**. Sensorimotor estimates may therefore provide a reliable source of the correlation between quantities and numerosities and, consequently, contribute to update the representation of magnitude in the human brain **(c)**.

Second, based on the paramount importance in development to learn about the environment through motor interaction (i.e., especially with the mouth and the hands), sensorimotor transformation is not doubt crucial in establishing the correlation between discrete and continuous magnitudes. For instance, the size of the mouth opening increases with the size of the handled object or of the edible food (Gentilucci et al., 2001). Similarly, the grip aperture is known to correlate with the size of the object to be grasped (Olivier et al., 2007; see Graphical Abstract 1b). Estimates used by the motor system to program manual or mouth movements may therefore represent a key mechanism subserving the statistical learning process (see Grade et al., 2016). In line with this view, not only the perception of numbers (Ranzini et al., 2011; Gianelli et al., 2012; Girelli et al., 2016; Namdar and Ganel, 2016), but also of different continuums that have a recurrent statistical mapping with visual size have been recently shown to interfere with motor planning and execution in adulthood. For example, the size and mass of an object tend to naturally correlate with its resonant frequency and loudness, with lower and louder sounds that are visually associated with larger rather than smaller objects (Spence, 2011). Critically, similar effects have been reported for action execution (Sedda et al., 2011; Rinaldi et al., 2016). Although there is evidence showing that even affordance, which refers to the activation of action patterns from perceived objects (Gibson, 1979), interferes with numerical processing (e.g., Badets et al., 2007; Ranzini et al., 2011), we believe that early in life real movements may be the primary source for grasping the “sense of magnitude.”

To sum up, despite renewed interest in how the body bootstraps learning over development (Smith and Gasser, 2005), the Leibovich et al.'s theoretical framework overlooks the role of sensorimotor experience in the refinement of numerical knowledge. Yet, as briefly reviewed, *acting* systematically on the environment directly enriches the natural correlation between numerosities and continuous quantities in the human mind (see Graphical Abstract 1c). Accordingly, it seems highly reasonable to suggest that the “sense of magnitude” develops on a self-enforcing activation loop between perception and action.

## Author contributions

LR wrote the first draft of the manuscript; both authors discussed it, critically revised it, and agreed on the final version.

### Conflict of interest statement

The authors declare that the research was conducted in the absence of any commercial or financial relationships that could be construed as a potential conflict of interest.

## References

[B1] Andres M. Olivier E. Badets A. (2008). Actions, words, and numbers: a motor contribution to semantic processing? Curr. Dir. Psychol. Sci. 17, 313–317. 10.1111/j.1467-8721.2008.00597.x

[B2] Badets A. Andres M. Di Luca S. Pesenti M. (2007). Number magnitude potentiates action judgements. Exp. Brain Res. 180, 525–534. 10.1007/s00221-007-0870-y 17279382

[B3] Bueti D. Walsh V. (2009). The parietal cortex and the representation of time, space, number and other magnitudes. Philos. Trans. R. Soc. B 364, 1831–1840. 10.1098/rstb.2009.0028 19487186 PMC2685826

[B4] Dehaene S. (1997). The Number Sense: How The Mind Creates Mathematics. New York, NY: Oxford University Press.

[B5] Feigenson L. Dehaene S. Spelke E. (2004). Core systems of number. Trends Cogn. Sci. (Regul. Ed). 8, 307–314. 10.1016/j.tics.2004.05.002 15242690

[B6] Frost R. Armstrong B. C. Siegelman N. Christiansen M. H. (2015). Domain generality versus modality specificity: the paradox of statistical learning. Trends Cogn. Sci. (Regul. Ed.). 19, 117–125. 10.1016/j.tics.2014.12.010 25631249 PMC4348214

[B7] Gandini D. Lemaire P. Dufau S. (2008). Older and younger adults' strategies in approximate quantification. Acta Psychol. 129, 175–189. 10.1016/j.actpsy.2008.05.009 18606394

[B8] Gebuis T. Reynvoet B. (2012). Continuous visual properties explain neural responses to nonsymbolic number. Psychophysiology 49, 1649–1659. 10.1111/j.1469-8986.2012.01461.x 23046492

[B9] Gentilucci M. Benuzzi F. Gangitano M. Grimaldi S. (2001). Grasp with hand and mouth: a kinematic study on healthy subjects. J. Neurophysiol. 86, 1685–1699. 11600632 10.1152/jn.2001.86.4.1685

[B10] Gianelli C. Ranzini M. Marzocchi M. Micheli L. R. Borghi A. M. (2012). Influence of numerical magnitudes on the free choice of an object position. Cogn. Process. 13, 185–188. 10.1007/s10339-012-0483-7 22806667

[B11] Gibson J. (1979). The Ecological Approach to Visual Perception. Boston, MA: Lawrence Erlbaum Associates, Inc.

[B12] Girelli L. Perrone G. Croccolo F. Roman E. H. Bricolo E. Mancin M.. (2016). Manual actions cover symbolic distances at different speed. Acta Psychol. 169, 56–60. 10.1016/j.actpsy.2016.05.002 27232553

[B13] Grade S. Badets A. Pesenti M. (2016). Influence of finger and mouth action observation on random number generation: an instance of embodied cognition for abstract concepts. Psychol. Res. 81, 538–548. 10.1007/s00426-016-0760-7 26927471

[B14] Henik A. Gliksman Y. Kallai A. Leibovich T. (2017). Size perception and the foundation of numerical processing. Curr. Dir. Psychol. Sci. 26, 45–51. 10.1177/0963721416671323

[B15] Leibovich T. Ansari D. (2016). The symbol-grounding problem in numerical cognition: a review of theory, evidence and outstanding questions. Can. J. Exp. Psychol. 70, 12–23. 10.1037/cep0000070 26913782

[B16] Leibovich T. Katzin N. Harel M. Henik A. (2016). From ‘sense of number’ to ‘sense of magnitude’ – the role of continuous magnitudes in numerical cognition. Behav. Brain Sci. [Epub ahead of print]. 10.1017/S0140525X16000960 27530053

[B17] Namdar G. Ganel T. (2016). The effects of magnitude on visually guided action and perception. J. Vis. 16, 453–453. 10.1167/16.12.453 26053340

[B18] Newcombe N. S. Levine S. C. Mix K. S. (2015). Thinking about quantity: the intertwined development of spatial and numerical cognition. Wiley Interdiscip. Rev. Cogn. Sci. 6, 491–505. 10.1002/wcs.1369 26415916

[B19] Olivier E. Davare M. Andres M. Fadiga L. (2007). Precision grasping in humans: from motor control to cognition. Curr. Opin. Neurobiol. 17, 644–648. 10.1016/j.conb.2008.01.008 18337084

[B20] Ranzini M. Lugli L. Anelli F. Carbone R. Nicoletti R. Borghi A. M. (2011). Graspable objects shape number processing. Front. Hum. Neurosci. 5:147. 10.3389/fnhum.2011.00147 22164141 PMC3230823

[B21] Rinaldi L. Lega C. Cattaneo Z. Girelli L. Bernardi N. F. (2016). Grasping the sound: auditory pitch influences size processing in motor planning. J. Exp. Psychol. Hum. Percept. Perform. 42, 11–22. 10.1037/xhp0000120 26280267

[B22] Sedda A. Monaco S. Bottini G. Goodale M. A. (2011). Integration of visual and auditory information for hand actions: preliminary evidence for the contribution of natural sounds to grasping. Exp. Brain Res. 209, 365–374. 10.1007/s00221-011-2559-5 21290243

[B23] Smith L. Gasser M. (2005). The development of embodied cognition: six lessons from babies. Artif. Life 11, 13–29. 10.1162/1064546053278973 15811218

[B24] Spence C. (2011). Crossmodal correspondences: a tutorial review. Atten. Percept. Psychophys. 73, 971–995. 10.3758/s13414-010-0073-7 21264748

[B25] Walsh V. (2003). A theory of magnitude: common cortical metrics of time, space and quantity. Trends Cogn. Sci. (Regul. Ed). 7, 483–488. 10.1016/j.tics.2003.09.002 14585444

[B26] Watson D. G. Maylor E. A. Bruce L. A. (2007). The role of eye movements in subitizing and counting. J. Exp. Psychol. Hum. Percept. Perform. 33, 1389–1399. 10.1037/0096-1523.33.6.1389 18085951

